# Presurgical Virtual Planning and Intraoperative Navigation with 3D-Preformed Mesh: A New Protocol for Primary Orbital Fracture Reconstruction

**DOI:** 10.3390/life14040482

**Published:** 2024-04-06

**Authors:** Giuseppe Consorti, Gabriele Monarchi, Lisa Catarzi

**Affiliations:** 1 Department of Maxillofacial Surgery, Azienda Ospedaliera Universitaria “Ospedali Riuniti di Ancona” Umberto I, 60126 Ancona, Italy; giuseppe.consorti@ospedaliriuniti.marche.it; 2 Department of Maxillofacial Surgery, Hospital of Perugia, Sant’Andrea delle Fratte, 06129 Perugia, Italy; gabriele.monarchi@gmail.com; 3 Department of Maxillofacial Surgery, University of Siena, 53100 Siena, Italy

**Keywords:** blowout fracture, orbital fracture, preformed mesh, orbital implant, navigation, virtual surgical planning, titanium mesh, orbital trauma, orbital floor fracture, computer-assisted surgery

## Abstract

Purpose: This pilot study aims to evaluate the feasibility and effectiveness of computer-assisted surgery protocol with 3D-preformed orbital titanium mesh (3D-POTM), using presurgical virtual planning and intraoperative navigation in primary inferomedial orbital fracture reconstruction. Methods: Between March 2021 and March 2023, perioperative data of patients undergoing surgery for unilateral inferomedial orbital fracture treated with 3D-POTM were analyzed. Presurgical virtual planning with a Standard Triangle Language file of preformed mesh was conducted using the mirrored unaffected contralateral side as a reference, and intraoperative navigation was used. The reconstruction accuracy was determined by: correspondence between postoperative reconstruction mesh position with presurgical virtual planning and difference among the reconstructed and the unaffected orbital volume. Pre- and postoperative diplopia and enophthalmos were assessed. Results: Twenty-six patients were included. Isolated orbital floor fracture was reported in 14 (53.8%) patients, meanwhile medial wall and floor one in 12 (46.1%) cases. The mean difference between final plate position and ideal digital plan was 0.692 mm (95% CI: 0.601–0.783). The mean volume difference between reconstructed and unaffected orbit was 1.02 mL (95% CI: 0.451–1.589). Preoperative diplopia was settled out in all cases and enophthalmos in 19 (76.2%) of 21 patients. Conclusion: The proposed protocol is an adaptable and reliable workflow for the early treatment of inferomedial orbital fractures. It enables precise preoperative planning and intraoperative procedures, mitigating pitfalls and complications, and delivering excellent reconstruction, all while maintaining reasonable costs and commitment times.

## 1. Introduction

Inferomedial orbital wall fractures are still controversial in terms of how they should be managed. Treatment of this type of fracture entails a reduction of fracture segments anatomically in order to restore orbital volume. Indications of surgical treatment include double vision caused by incarceration of the orbital muscles or endo-orbital tissue, as documented by forced duction examination and as indicated by computed tomography (CT) scans; extensive fractures that result in enophthalmos and can cause dystopia. A fracture of the orbital wall is one of the most common in patients who have suffered facial trauma, and its incidence ranges from 18% to 50% of all craniomaxillofacial injuries [1,2].

During orbital reconstruction, complex anatomical structures must be preserved as well as vital structures such as the optic nerve. Especially when dealing with orbital wall fractures near the optic canal, failure to properly place orbital reconstruction material due to the concern for severe postoperative complications like blindness can result in suboptimal outcomes [3]. Precise dissection and proper placement of orbital reconstruction material are essential when addressing orbital wall fractures near the optic canal. With the development of imaging tools, alloplastic materials, and surgical techniques, adequate reconstructions have been possible for injured orbits [4].

A corrective orbital three-dimensional anatomy reconstruction is necessary due to the important role shape and symmetry play in eyeball projection and position. Despite variations in orbital conformation that may make orbital repair difficult, the current development of orbital titanium meshes, image management software as virtual presurgical planning, and intraoperative navigation technologies have significantly improved surgical strategies in orbital reconstruction [5,6]. First, the 3D-formed meshes have demonstrated their capacity to possess the appropriate shape, ensuring precise restoration of the anatomy of the orbital wall, whether it involves fractures of the orbital floor with or without involvement of the medial wall [7,8]. Second, the virtual surgical planning makes possible a direct three-dimensional preoperative presurgical planning, going to reconstruct the volume and walls of the fractured orbit through mirroring with the healthy orbit, and also allows us to virtually position the reconstructive plate by evaluating any excess portion to be cut intraoperatively. Finally, the introduction of surgical navigation in cranio-maxillofacial surgery radically changed the surgical approach to facial and orbital diseases [9].

In navigation-assisted surgery, the intraoperative position of instruments is coordinated with CT imaging of the patient’s anatomy during surgery. The main advantage of navigation is that the surgeon can instantaneously determine the position of the surgical instrument on the CT images and see, during the operation, if the reconstruction is performed according to presurgical planning. The integration of different technologies, particularly software and surgical navigation, opens new horizons for tailoring the reconstruction for each patient [10]. Though computer-based diagnosis and craniomaxillofacial surgery have been proposed in several situations, they have not yet been widely adopted for primary orbital fracture reconstruction [11]. Almost all of the cases reported in the literature are associated with delayed orbital reconstruction and are often linked to patient-specific implants, which have high costs and require external institutions for design and fabrication [12].

In this pilot study, we investigated the feasibility and clinical value of our computer-assisted surgery workflow with 3D-preformed orbital titanium mesh using presurgical virtual planning and intraoperative navigation for primary inferomedial orbital fracture reconstruction, analyzing the results.

## 2. Materials and Methods

### 2.1. Study Design

Clinical data of patients who underwent primary reconstruction for unilateral inferomedial orbital fractures, treated with a computer-assisted surgery protocol utilizing 3D-preformed orbital titanium mesh (3D-POTM), at the University Hospital Ospedali Riuniti Ancona, Italy, between March 2021 and March 2023, were retrospectively collected and analyzed. No alternative reconstruction methods were employed at the authors’ institution during the study period.

Inclusion criteria comprised patients aged 18 or older at the time of the operation, with an orbital defect exceeding 2 cm^2^ on CT scans, presenting either a unilateral isolated orbital floor fracture or a combination of unilateral orbital floor and medial wall fractures, involving the buttress of the transition zone. Additionally, eligible patients had available preoperative and postoperative clinical and medical records.

Patients with fractures associated with vision loss in the affected eye, those requiring urgent orbital decompression, or those with craniofacial malformations leading to orbital asymmetries were excluded from the study.

All patients underwent physical examinations preoperatively, postoperatively, and during a follow-up period of at least 3 months. All operations were performed by the same surgeon (GC), a specialist in oral and maxillofacial surgery with more than 10 years of experience in orbital reconstruction.

### 2.2. Virtual Surgical Planning and Modification of 3D-Preformed Mesh

High-resolution CT with 0.6 mm slice imaging was performed on all patients.

The Digital Imaging and Communications in Medicine (DICOM) scan data derived from the preoperative CT scans were subsequently converted into an STL (Standard Triangle Language) file. Using the Brainlab Elements Contouring software package (version 4.0, Brainlab, Feldkirchen, Germany), the STL file underwent processing to mirror the unaffected orbit onto the affected one, allowing the creation of a virtual reconstruction of the affected orbital region.

In all cases, the 3D-preformed titanium mesh, available in large or small plate variants with a thickness of 0.4 mm (3D Orbital Floor Plate, Stryker Corp., Kalamazoo, MI, USA), was utilized. The choice between the large or small plate was made based on the size of the orbital defect.

**Figure 1:**
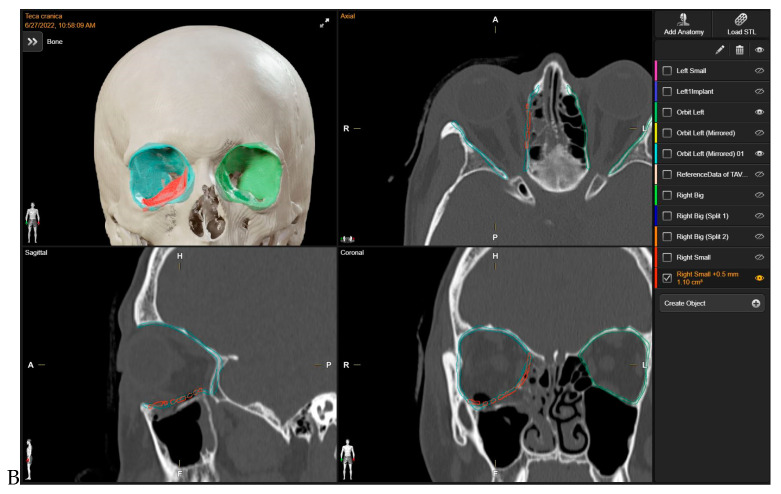
Preoperative Virtual Planning. The 3D-preformed titanium mesh’s STL files were virtually overlaid to align with the “ideal orbital reconstruction” achieved by using the mirrored unaffected contralateral side as a reference. In red: STL files for 3D-preformed titanium mesh. Light blue: mirrored orbit. Green: safe orbit.

Initially, mirroring techniques were employed to achieve a virtually ideal reconstruction of the orbital defect. Subsequently, the STL file of the plate was uploaded into the Brainlab Element Software 4.0. The 3D-POTM’s STL files were digitally overlaid onto the simulated optimal 3D virtual orbital reconstruction. The software’s tools were then used to manipulate the plate object, adjusting its position within the reconstructed orbit created via mirroring. To ensure a precise fit to the boundaries of the mirroring, the “object management” tool was employed to cut the plate. This process allowed for the customization of the plate’s shape, ensuring optimal adaptation to the specific orbital defect and the mirrored orbit. In this stage, digital planning was employed to determine the required trimming of surplus mesh components, and the ideal placement for the implant was computed by two senior surgeons. This assessment drew upon the data from the STL file and preoperative scans analyzed through Brainlab Elements Contouring, version 4.0 (Brainlab, Feldkirchen, Germany). In contrast, a bending plate was not designed. The preformed meshes utilized in the study (3D Orbital Floor Plate, Stryker Corp., Kalamazoo, MI, USA) were already shaped appropriately to accurately reproduce the orbital floor and/or the transition zone between the orbital floor and medial orbital wall, without requiring bending. The estimation and verification of the implant’s location within the orbit, as well as its connections with the anatomical structures of the orbit, were subsequently confirmed (Figure 1). In all cases, presurgical VSP took place before the patient’s hospitalization, primarily in the outpatient clinic. The process of virtual planning, including the manipulation of 3D models and the determination of surgical approaches, was conducted before the hospitalization. The navigation system (Brainlab Curve, Brainlab, Munich, Germany) was loaded with both the preoperative CT scan and the STL file of the 3D-preformed implant.

### 2.3. Reconstruction of the Orbital Walls and Operative Treatment

All patients were treated under general anesthesia. The surgical approach utilized was the transconjunctival preseptal approach, combined with a medial transcaruncular incision in instances where there were fractures of the orbital floor and medial wall. Cantolysis/cantotomy procedures were conducted as required to attain adequate access to extensive orbital floor and medial wall fractures.

The implant meshes were placed within the orbit, rotating from lateral to medial, and accompanied by anteroposterior sliding movements to naturally find their position. During the operation, the surgeon sought this position, considering it the “best fit” for the implant.

Intraoperatively, the patient’s positioning and anatomical features were aligned with the preoperative plan using a magnetic-based navigation system and specific landmarks.

Intraoperative navigation (Brainlab Curve, Brainlab, Munich, Germany) was utilized to verify the implant positioning and ensure real-time confirmation of accurate placement, thereby adhering to the boundaries outlined in the mirrored presurgical plan, which represents the ideal outcome.

Additionally, four points were designated as checkpoints during surgery to optimize implant placement: the inferior orbital margin, the inferior orbital fissure, the posterior bony ledge (orbital process of the palatine bone), and the transition buttress between the orbital floor and medial wall.

Postoperative CT scans were performed on all patients, typically within 1 to 3 days following surgery, while the patient was still hospitalized.

### 2.4. Study Variables and Outcomes

We analyzed the results of the computer-assisted surgery protocol with 3D-preformed orbital titanium mesh, incorporating presurgical virtual planning and intraoperative navigation for primary inferomedial orbital fracture reconstruction.

The reconstruction accuracy was assessed based on the correspondence between the postoperative reconstruction mesh position and the presurgical virtual planning.

Additionally, we measured the difference in volume between the reconstructed and unaffected orbital structures.

**Figure 2:**
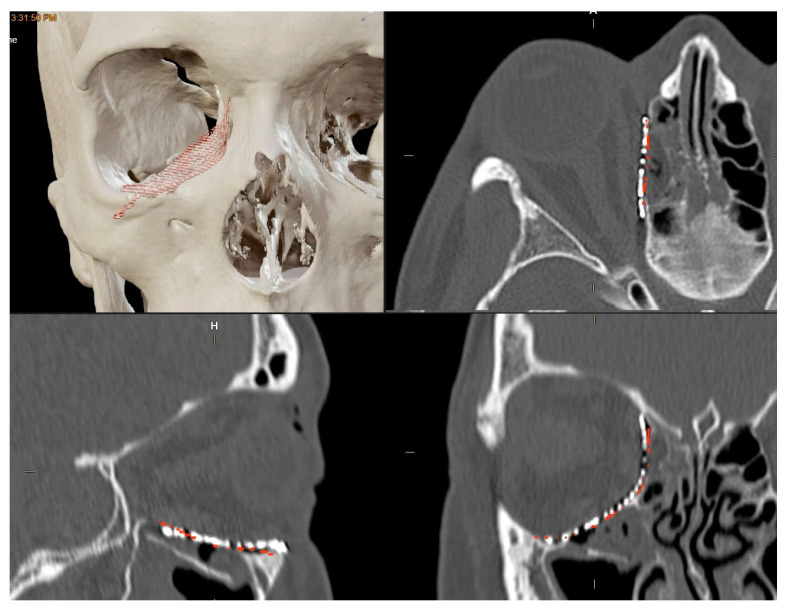
The overlaying of postoperative CT scans onto preoperative planning images.

To evaluate the correspondence between the postoperative CT scan and the Virtual Surgical Planning (VSP), the process of overlaying postoperative CT scans onto preoperative planning images was executed through the “Image Fusion” tool within the software. “Brainlab Elements Contouring”, version 4.0 (Brainlab, Feldkirchen, Germany) (Figure 2), which creates an automatic alignment of preoperative images and postoperative CT scans. Subsequently, through visual inspection, areas where the boundaries of the superimposition were not perfectly aligned were carefully examined. In these regions, the mismatch between the postoperative CT plate and the simulated virtual positioning of the mesh was manually measured.

This manual calculation involved identifying points of deviation between the planned and actual positions and measuring the maximum difference observed. This difference, termed the “maximum error”, was then recorded for further analysis.

Orbital volume measurements of both the reconstructed and unaffected orbits were conducted using a dedicated tool for orbital volume measurement integrated into the software suite “Brainlab Elements Contouring”, version 4.0 (Brainlab, Feldkirchen, Germany). To maintain measurement integrity and minimize potential bias, we relied on the automatically generated volumes produced by the software. Given the precision and specialized functionality of this tool within the Brainlab portfolio, we considered the automatically generated volumes to be robust and indicative of the true orbital volumes. By calculating boundaries on presurgical CT scans, the software’s algorithms incorporate anatomical landmarks and contours, ensuring accurate delineation even in cases of anatomical variation or pathology, without the need for manual adjustments.

Through utilization of the automated volumes generated by the Brainlab software, our objective was to reduce potential bias and ensure consistency in measurements, thereby enhancing the reliability of our findings.

**Figure 3:**
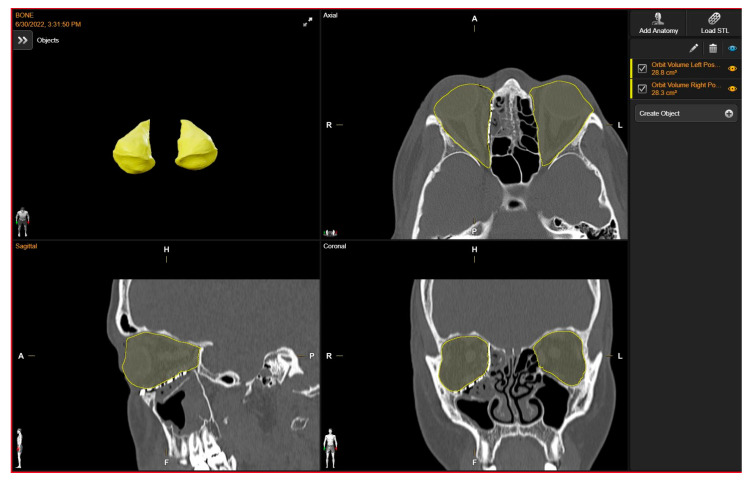
Post- operative Orbital Volume Measurements.

The volume of the unaffected contralateral orbit serves as a reference for comparison. Subsequently, the volume discrepancy between the reconstructed and contralateral orbits was computed (Figure 3).

Clinical findings, including enophthalmos and the presence of diplopia, were evaluated preoperatively and 6 months after surgery. Enophthalmos was assessed using a Hertel exophthalmometer, with values below 14 mm indicating enophthalmos. Specifically, the authors consider the clinically significant postoperative enophthalmos with a difference between the affected orbit and safe orbit > 2 mm.

Furthermore, CT scans were employed to complement our assessment. It is worth noting that a 1 cm^3^ increase in orbital volume typically results in approximately 0.8 mm of enophthalmos, as previously reported [13]. Surgical details including the timing of VSP, timing between trauma and operative treatment, approach, as well as complications were documented. Intraoperatively, the duration of both the actual insertion of the implant and the modification of the plate was assessed, as well as the overall operating time. The recording began after the fracture was fully exposed and prepared for the insertion of the orbital mesh. Time recording concluded when the mesh achieved its final shape and position without the need for additional corrections.

### 2.5. Statistical Analysis

The Kolmogorov–Smirnov test was employed to test normal distribution. Categorical variables were presented as frequencies and percentages, whereas continuous variables were described using mean and standard deviation. The statistical analysis was performed using SPSS software (version 12.0; SPSS Inc., Chicago, IL, USA) on a Windows platform.

## 3. Results

### 3.1. Population Characteristics

The study comprised 26 patients with an average age of 42.5 years (SD = 16.8). Of these, 18 were male (69.2%) and 8 were female (30.7%). In total, 14 (53.8%) patients exhibited orbital floor fractures, while 12 (46.1%) patients presented with fractures involving both the orbital floor and medial wall. Patient demographics and fracture classifications are outlined in Table 1. All 26 patients were followed up for at least 3 months.

### 3.2. Reconstruction Accuracy

Postoperative CT scans demonstrated precise fitting of the orbital mesh plates. Overlaying postoperative 3D CT images onto preoperative virtual reconstruction images revealed a fitting accuracy with a maximum error of 1.3 mm. The mean difference between the final plate position and the ideal digital plan was 0.692 mm (95% CI: 0.601–0.783).

Analysis of post-treatment orbital volume indicated that volume differences between the reconstructed and unaffected orbits ranged from −2.3 mL to 0.7 mL. The mean volume difference between the reconstructed and unaffected orbits was 1.02 mL (95% CI: 0.451–1.589). All these results are summarized in Table 2.

### 3.3. Diplopia and Enophthalmos

In the preoperative course, 23 (88.5%) patients experienced diplopia and 21 (80.7%) enophthalmos. All patients experiencing preoperative diplopia reported complete resolution three months post-surgery. Enophthalmos was effectively resolved in 19 patients (76.2%). Remarkable clinical outcomes, including significant changes in globe projection, were achieved in all patients. The evaluation of preoperative and postoperative diplopia and enophthalmos is outlined in Table 2. No major postoperative or intraoperative complications were observed, and no revision surgery was necessary.

### 3.4. Surgical Approach, Timing, and Details

All cases were performed by the same surgeon possessing over a decade of expertise in orbital surgery. Preoperative virtual surgical planning took approximately 15 min. The mean time from trauma to surgery was 4.3 days (SD = 1.80). Patients’ hospitalization ranged from 2 to 5 days, varying according to individual patient needs and the complexity of the surgical procedure. Regarding the chosen surgical approach, the preseptal transconjunctival approach was selected in 18 (69.2%) cases, while a combination of the medial transcaruncular and preseptal transconjunctival approaches was employed in 7 (27.0%) cases. Cantotomia/cantolysis procedures were performed in 1 (3.8%) case. The 3D-preformed mesh cutting was necessary in 18 (66.6%) patients. Among these, the meshes were intraoperatively modified as per virtual programming in 16 (88.9%) cases, with only 2 (11.1%) cases requiring additional or alternative intraoperative modifications. The average operative time required was approximately 45 min.

The intraoperative mesh cutting time required was approximately one minute. Not one of the 3D plates was bent. The duration of the actual insertion of the implant was 4 min. All mesh placements were guided by magnetic-based navigation to ensure precise control over the shape and positioning of the implants. In the majority of cases, screws were not used to fix the implant. However, in instances where screws were utilized, they were typically placed on the outer surface of the inferior orbital rim. No implant dislocation or other major complications were observed.

## 4. Discussion

Based on the existing literature, fully computer-assisted surgery in orbital reconstruction is almost exclusively described and utilized with patient-specific implants. On the contrary, the latest technology used with preformed mesh is represented by the sole intra-navigation system. The present pilot study evaluated the results of a full computer-assisted surgery workflow with 3D-preformed orbital titanium mesh in primary orbital reconstruction. Our study showcased that through presurgical virtual planning and intraoperative navigation, the utilization of 3D-preformed titanium meshes resulted in highly accurate orbital reconstruction, effectively restoring orbital volume and ensuring optimal mesh positioning, thereby yielding favorable clinical outcomes.

The orbits resemble quadrangular pyramids with an anterior base, an apex, a trunk, and a rear; therefore, the posterior third is relatively small in volume. To ensure the function and aesthetic of the orbital region, proper reconstruction of the orbital walls is essential. Surgical surgeons have always strived to obtain the best orbital wall reconstruction using the best materials, with the final result being dependent on their own skill [14,15]. To correct the eyeball’s position or reconstruct the orbital walls, various surgical techniques have been proposed. Until now, precisely reconstructing the exact anatomy of the orbital cavity has always represented a topic of study and debate [16]. To increase the precision and safety of orbital reconstruction, new surgical technologies have been popularized, such as anatomically shaped implants, computer-assisted surgical planning, and surgical navigation [17,18]. There have been several recent modifications to the computer-based craniomaxillofacial surgical method. As opposed to conventional surgery, image-guided surgery utilizes preoperative imaging data to plan the surgical procedure, and the surgery is guided by a surgical navigation system during surgery.

Surgical procedures and preoperative planning have been refined to produce better clinical outcomes [19]. As a result of this experience, we started applying preoperative planning and navigation systems to the reconstruction of orbital fractures. Although navigation-assisted orbital reconstructions offer many advantages, there are few cases described in the literature [20]. Several authors have raised the importance of anatomically precise reconstructions of the orbital walls, including the most posterior parts of the orbit [21]. Thanks to the intraoperative navigation system, we were able to visualize the position of a deep hidden area close to the posterior surfaces of the medial and inferior orbital walls close to the optic nerve during the dissection [22]. It is advantageous to have these characteristics to perform surgical operations both safely and accurately. To date, computer-assisted navigation surgery for orbital reconstruction has not been routinely performed in the international literature. Some reports, however, describe the use of the navigation system to correct post-traumatic orbital deformities [23,24]. For all previous studies that used the navigation system for orbital reconstruction, delayed corrections were performed.

The present study involved the early correction of fractures of the inferior and/or medial walls of the orbit in 26 patients. In these cases, it is mandatory to obtain accurate coverage of the orbital defect after appropriate reduction of the herniated orbital tissue. Patients in this group had surgery after the swelling in their soft tissues reduced to a certain extent, but it had not completely subsided. An increase in intraorbital pressure would result from restoring herniated orbital fat despite residual soft tissue swelling in the orbit [25,26]. A constant focus is being placed on the advancement of orbital titanium meshes, preoperative planning software, and intraoperative navigation methods [5].

Despite the increasing popularity of patient-specific orbital implants, preformed meshes continue to be the most popular choice for treating orbital fractures in adults, while bone grafts and absorbable materials are often preferred in children [27]. Furthermore, the 3D-preformed orbital titanium meshes have demonstrated their ability to effectively address the anatomy of the orbital walls, providing comprehensive coverage for orbital floor reconstructions, whether with or without fractures involving the medial wall. They are manufactured to mimic the correct shape, accurately reproducing the anatomy of the orbital wall, including both the orbital floor and medial wall, along with a predesigned posterior retrobulbar bulge [28]. In our study, in alignment with findings reported in the literature [29], our observation revealed that 3D-preformed plates (specifically, the 3D Orbital Floor Plate, available in both large and small sizes, with a thickness of 0.4 mm) were sufficient to completely address orbital floor defects or inferomedial orbital fractures, even in cases of considerable extent, yielding favorable outcomes [30,31]. The use of preformed meshes appears to be a promising method for restoring the orbit’s unique shape precisely.

In order to achieve optimal results, it seems that the surgeon’s decision to position and design the plate is crucial. We reported a maximum error of 1.3 mm in superimposition between postoperative CT and preoperative virtual reconstruction images with a median difference between final plate position and preoperative planning of 0.692 mm (95% CI: 0.601–0.783). The recorded post-treatment orbital volume showed a difference ranging from −2.3 mL to 0.7 mL between virtual reconstruction and postoperative CT. The mean volume difference between the reconstructed and unaffected orbits was 1.02 mL (95% CI: 0.451–1.589). In the authors’ experience, bending 3D-preformed meshes is unnecessary and may result in errors. To accurately recreate the anatomy of the orbital floor and medial wall, these meshes are designed in the correct shape. Sometimes, however, the preformed plates may be in excess of the healthy bone margins surrounding the fracture. In this case, there is a risk of redundancy of the titanium plate, representing a potential source of error, or it could be necessary to spend a significant amount of time refining redundant parts of the mesh based on the assessed defect size to achieve accurate orbital defect reconstruction. The computer-assisted surgery workflow can help with this. The surgeon can simplify the intraoperative cutting process by planning the plate’s cutting during virtual preparation, with very high precision, designing the plate based on fracture defect and based on mirrored orbit reducing the need for extensive adjustments during surgery. The efficiency and feasibility of incorporating virtual presurgical planning into the overall surgical workflow are underscored by the fact that it took only 15 min to carry it out. Despite the relatively minimal time investment, these sessions can yield significant benefits in terms of intraoperative efficiency and surgical outcomes. As a result of adequate preoperative planning, the orbital plate had to be shaped and sized precisely to match the defect, allowing the orbital wall to be corrected. The possibility of loading the STL file of the preformed plate at the level of the virtual design program and superimposing it on the bone defect recreated starting from the patient’s CT scan made it possible to calculate the excess of the preformed titanium plate and study which portion of the same be sectioned to ensure the perfect bony fit. The elimination of major manipulations, such as repetitive implant fittings, not only reduces operative time but also reduces potential soft tissue damage around the periorbital area and the potential overall complications. By utilizing 3D-preformed mesh and the computer-assisted protocol, orbital fractures can be fully “in-house” restored. In addition to being easily reproducible, it eliminates the need for engineering expertise to design and process the implant. In addition, the procedure is self-sufficient and not constrained by delivery times. The optimal position and shape of the implant could be calculated by uploading the 3D-preformed mesh’s STL file and the preoperative CT into the virtual planning software. Of the 26 patients who came to our observation and were included in the study, 23 patients out of 26 (88.5%) reported diplopia and 21 (80.7%) enophthalmos in the preoperative period. As regards complications or sequelae reported postoperatively, all patients who experienced preoperative diplopia reported complete resolution three months after surgery. Additionally, enophthalmos was successfully resolved in 19 patients (76.2%). Going to analyze the surgical technique applied and operating times, 3D-preformed mesh cutting was necessary for 18 (66.6%) of the patients. Among these cases, the meshes were intraoperatively modified as per virtual programming in 16 (88.9%) instances, with only 2 (11.1%) requiring further or different intraoperative modifications to ensure a better fitting of the same to the patient’s skeleton. The overall operative time required for intraoperatively cutting the plates was extremely limited, with an average of approximately 1 min and a time of approximately 4 min for insertion at the surgical site. No bending of any preformed plate was performed. All mesh placements were facilitated using magnetic-based navigation to precisely control both the shape and position of the implants. Intraoperative navigation systems, as well as intraoperative CT scans, play pivotal roles in orbital fracture repair, each offering distinct advantages. Both provide real-time imaging, enabling surgeons to precisely assess the shape and position of implants during the procedure. Nowadays, intraoperative CT is more commonly used. However, it comes with a high cost and presents potential radiation exposure associated with repeated scans, posing challenges. On the other hand, intraoperative navigation systems offer a cost-effective alternative, aiding in precise implant placement without the need for continuous imaging and the requirement of a radiologic technician in the operating room, thus reducing the overall duration of the procedure. While both technologies contribute to improved surgical outcomes, intraoperative navigation systems may be particularly advantageous in orbital fracture repair due to the intricate nature of the procedure and the need to maintain a limited visual field. Thus, the choice between intraoperative CT scans and navigation systems depends on various factors. Obviously, there was a learning curve for the initial phase of the surgery with the use of the virtual simulation program, with the preformed orbital plate counting, and with the navigation system.

Orbital fractures represent the most challenging fractures of the facial skeleton to treat due to their complexity, the anatomical area involved, management requirements, and the lack of validated protocols. The authors have introduced a new approach to these fractures by utilizing new technologies such as intraoperative navigation and computerized programming using 3D-preformed mesh. These promising findings suggest that the utilization of 3D-preformed mesh plates could yield excellent outcomes for the reconstruction of inferomedial orbital fractures, offering the additional benefits of cost and time savings [32].

### Limitations

Several limitations should be acknowledged when interpreting the results of this study. Firstly, the retrospective design may have introduced inherent selection bias, potentially limiting the robustness of our findings. Secondly, the relatively small sample size may also affect the generalizability of the results. However, the results obtained in this preliminary study, despite the relatively small number of cases, provide a basis for validating this technique on a larger scale. Future research of high quality is encouraged to validate our findings and substantiate their applicability in a larger patient cohort.

Furthermore, this study lacked a control group of patients who underwent alternative surgical techniques; however, this omission did not affect the primary objectives of the study. Lastly, it is important to note that this study was conducted at a single center in Italy, thus limiting the generalizability of the findings to broader populations. Despite the acknowledged limitations, it is crucial to highlight a significant strength of this study, which proposes an effective and straightforward computer-assisted protocol utilizing 3D-preformed mesh for the primary reconstruction of inferomedial orbital fractures.

## 5. Conclusions

This clinical study demonstrates that blowout fractures can be safely treated through the positioning of 3D-preformed and contoured orbital plates, guided by 3D programming and a surgical navigation system.

The described computer-assisted workflow facilitates accurate preoperative planning and intraoperative cutting and positioning of the plate, avoiding pitfalls and complications. It offers excellent reconstructive results for inferomedial orbital fractures, along with reduced costs and time commitments.

## Figures and Tables

**Table 1:** Patient demographics.

Characteristic	n (%)
Sex	
Male	18 (69.2%)
Female	8 (30.7%)
Fracture Type	
Orbital Floor and Medial Wall	12 (53.8%)
Isolated Orbital Floor	14 (46.1%)

**Table 2:** Postoperative outcomes.

Accuracy of Orbital Reconstruction	Range	Mean
Postoperative vs. Virtual Surgical Planning Mesh Position	1.3–0.4 mm	0.69 mm (95% CI: 0.601–0.783)
Orbital Volume Different between reconstructed vs. unaffected orbit	−2.3 mL to 0.7 ml	1.02 mL (95% CI: 0.451–1.589)
**Postoperative Clinical Findings**	**n (%)**	
Diplopia	0 of 23 (0%)	
Enophthalmos	2 of 19 (23.8%)	
**Major Complication**	0 (%)	

## Data Availability

Additional details regarding the data supporting the reported results can be made available upon request to the authors.
